# Identification and Molecular Characteristics of a Novel Single-Stranded RNA Virus Isolated from Culex tritaeniorhynchus in China

**DOI:** 10.1128/spectrum.00536-23

**Published:** 2023-06-26

**Authors:** Zhen Wu, Jingyu Liu, Xiuwei Feng, Yuli Zhang, Lin Liu, Guoyu Niu

**Affiliations:** a School of Public Health, WeiFang Medical University, Weifang, China; b Yantai Center for Disease Control and Prevention, Yantai, China; c Immune-Path Biotechnology (Suzhou) Co., Ltd., Suzhou, China; Centro de Investigacion y de Estudios Avanzados del Instituto Politecnico Nacional

**Keywords:** Hubei mosquito virus 2, *Culex tritaeniorhynchus*, growth characteristics, Shandong province, molecular biology, evolution, virology

## Abstract

Hubei mosquito virus 2 (HMV2) is a novel mosquito virus that was first identified in 2016 in Hubei Province, China. Until now, HMV2 has been shown to be endemic in some areas of China and Japan, but its biological characteristics, epidemiology, and pathogenicity are not yet known. This report describes the detection of HMV2 in mosquitoes that were collected in Shandong Province in 2019 and presents the first isolation and molecular characterization of the virus. In this study, a total of 2,813 mosquitoes were collected and then divided into 57 pools, according to location and species. qRT-PCR and nested PCR were performed to confirm the presence of HMV2, and its genomic features, phylogenetic relationships, growth characteristics, and potential pathogenicity were further analyzed. The results showed that HMV2 was detected in 28 of the 57 mosquito pools and that the minimum infection rate (MIR) for HMV2 was 1.00% (28/2,813). A HMV2 strain and 14 viral partial sequences were obtained from the HMV2-positive pools, including one complete genome sequence. A phylogenetic analysis revealed that HMV2 from Shandong Province shared over 90% identity with other reported isolates and was closely related to the Culex inatomii luteo-like virus.

**IMPORTANCE** Our study provided important epidemiological evidence for the epidemic of HMV2 in Shandong Province. Here, we report the first isolation and molecular characteristics of this virus and enrich our knowledge of the distribution of HMV2 in China.

## INTRODUCTION

Mosquitoes are blood-sucking dipterans belonging to the Culicidae family (1). There are currently more than 3,500 species of mosquitoes distributed in all parts of the world, except Antarctica. They are considered to be important vectors of disease transmission and can carry a variety of viruses (2). Viruses transmitted by mosquitoes are mainly divided into two categories: mosquito-specific viruses and mosquito-borne viruses (3). A mosquito-specific virus is a mosquito-infecting virus that replicates in mosquito cells *in vivo* and outside the body but does not infect humans or vertebrates (4). These include the cell-fusing agent virus (CFAV) (5), Kamiti River Virus (KRV) (6), and Culex flavivirus (CxFV) (7), among others. In contrast, mosquito-borne viruses are a class of viruses that not only can replicate in mosquito cells but also can be transmitted to humans or vertebrates and may cause disease outbreaks in human or animal populations, such as Yellow fever virus (YFV), Dengue virus (DENV) (8), Chikungunya virus (CHIKV) (9), and Zika virus (ZIKV) (10). In recent years, outbreaks of these mosquito-borne viruses have led to large numbers of human and animal infections and deaths, causing an incalculable loss to humans. Therefore, people have stepped up their monitoring of mosquitoes as well as the research of mosquito-borne viruses (11).

At the same time, the development of metagenomic sequencing technology has promoted the detection and monitoring of mosquito viruses, thereby resulting in the discovery and research of a large number of new mosquito viruses, including Getah virus (12), Ross River virus (13), Banna orbivirus (14), and many others. This provided new insights into the complexity and diversity of the viruses that are carried by mosquitoes, and these had important implications for the dynamic surveillance of pathogens and the responses to emerging and reemerging infectious diseases (15).

HMV2, belonging to Riboviria, is a single-stranded RNA virus that was first detected in mosquitoes from Hubei Province, China, in 2016 (16). Subsequently, HMV2 was detected in Armigeres subalbatus, Anopheles sinensis, Aedes aegypti, and Culex tritaeniorhynchus in Yunnan Province, China, as well as in *Culex tritaeniorhynchus* in Ishikawa and Tottori, Japan (12, 17). However, as a novel mosquito-borne virus, its pathogenicity and molecular characteristics have not yet been elucidated. In this study, a strain of HMV2 named 2019-LZ-13 was isolated from *Culex tritaeniorhynchus* collected from Shandong Province, China, in 2019, which was the first report of a successful isolation of HMV2. A phylogenetic analysis showed that the HMV2 was closely related to the *Culex inatomii* luteo-like virus (CiLLV). In addition, HMV2 could proliferate stably in different mammalian cells, suggesting that mammals may act as their amplification hosts or intermediate hosts. Our study expanded the understanding of the evolutionary relationships and characteristics of HMV2 while also providing molecular evidence for the prevalence of HMV2 within Shandong Province.

## RESULTS

### Mosquito collection and viral nucleic acid detection.

**FIG 1:**
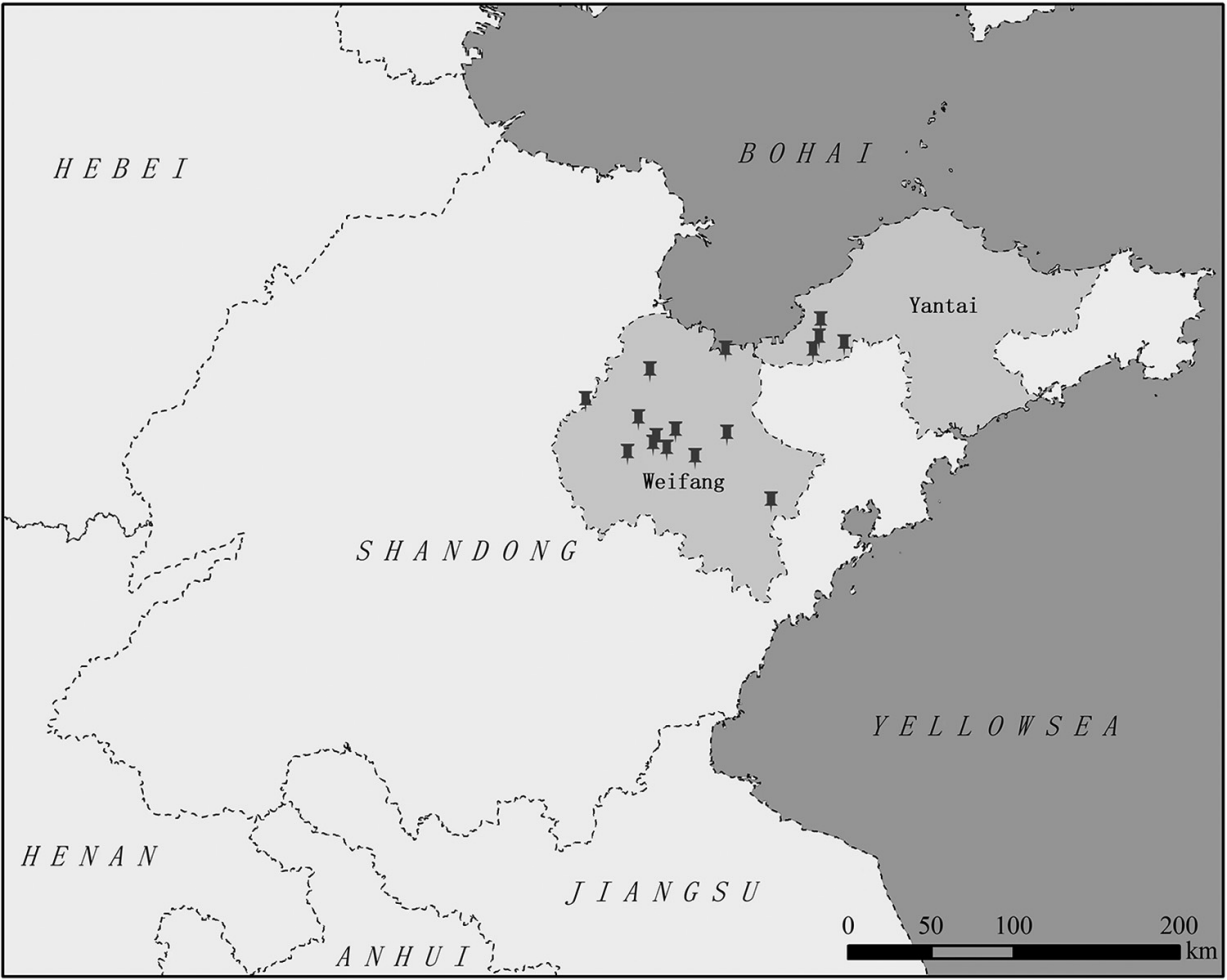
Geographic locations of the mosquito capture sites that were used to detect HMV2 infections in mosquitoes in Shandong Province, China, in 2019.

A total of 2,813 female mosquito samples were collected, including 365 Culex quinquefasciatus, 398 Aedes albopictus, 324 *Anopheles sinensis*, 402 *Armigeres subalbatus*, and 1,324 *Culex tritaeniorhynchus*. *Culex tritaeniorhynchus* (47.1%) was the dominant species, and it was followed by *Armigeres subalbatus* (14.3%). Geographically, 1,125 mosquitoes were collected from Weifang City, and 1,688 mosquitoes were collected from Yantai City (Fig. 1). The mosquitoes were divided into 57 mosquito pools, according to their sampling location and species. Of the 5 mosquito species that were detected, only *Culex tritaeniorhynchus* and Culex quinquefasciatus were positive for HMV2, and the MIR values were 1.74% (23/1,324) and 1.37% (5/365), respectively. No significant difference was found in the prevalence of HMV2 between the two mosquitoes. Meanwhile, the MIR values of HMV2 in mosquitoes from Weifang City and Yantai City were 0% and 1.66%, respectively.

### Virus isolation and electron microscopy.

**FIG 2:**
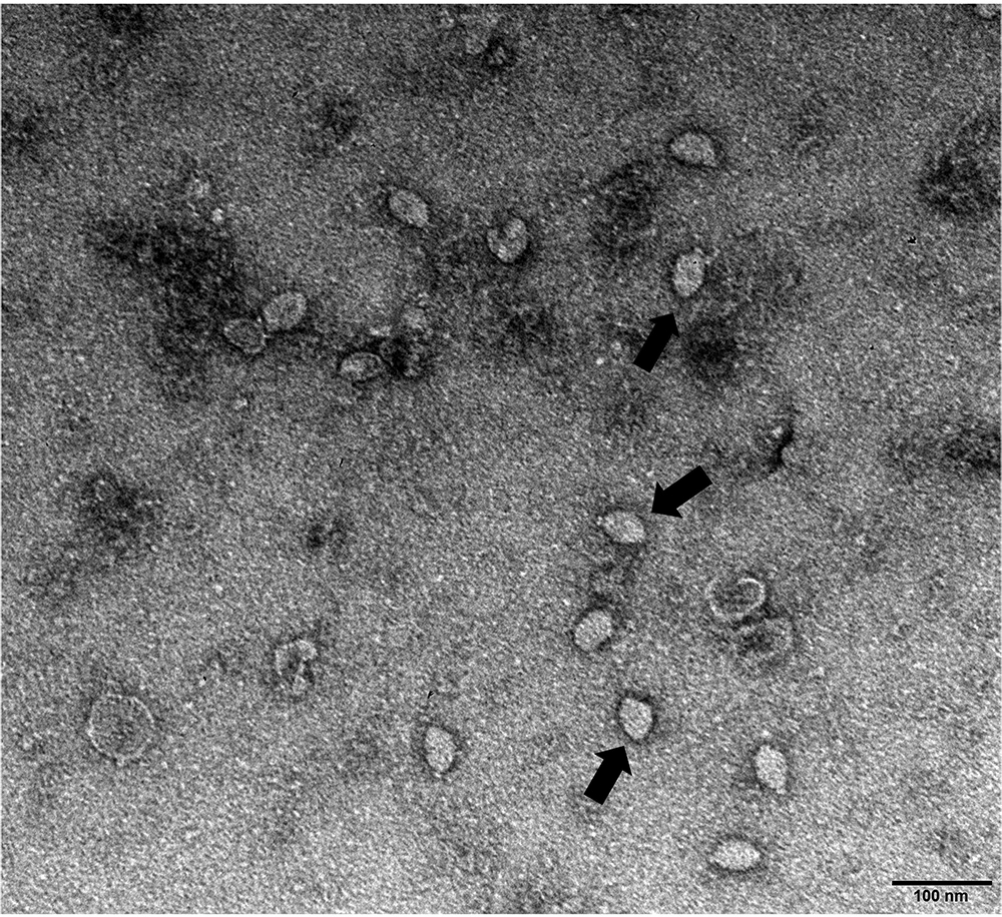
Electron micrograph of HMV2 particles. The black arrow refers to the HMV2 virion. The scale bars indicate a length of 100 nm. Samples of inoculated C6/36 cells were collected from July to August of 2019. After infected C6/36 cells underwent three passages, the supernatant of the third passage cells were collected for electron microscopic observations of viral particles.

The HMV2 strain named 2019-LZ-13 was isolated from one pool of mosquitoes (*Culex tritaeniorhynchus*) captured in Yantai City, Shandong Province, China. No CPE was found in C6/36 cells, even after three blind passages. Virus particles were observed in the supernatant of infected C3/36 cells via transmission electron microscopy. Spindle shaped and nonenveloped virus particles were observed in the particle after ultracentrifugation. The particles were approximately 55 nm in length and 40 nm in width. Such virions were evenly distributed in the field of vision, and this virion was not found in C6/36 cells that were not infected with the virus (Fig. 2).

### Growth characteristics of the virus in different cells.

**FIG 3:**
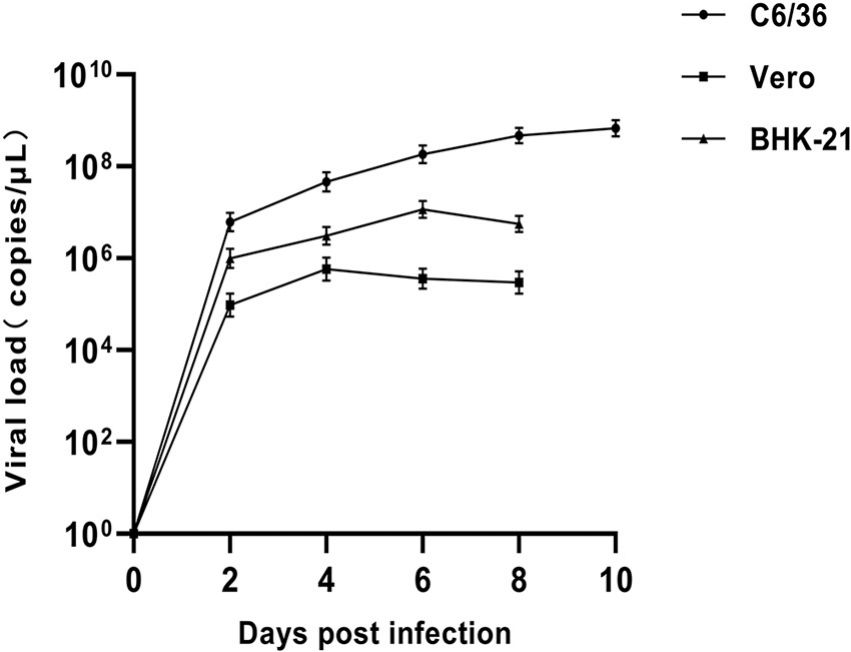
Growth kinetics of HMV2 in C6/36 cells, Vero cells, and BHK-21 cells. Vero, BHK-21, and C6/36 cells were inoculated with strain 2019-LZ-13. Viral titers were measured at different time points, and the experiment was repeated three times. The data represent the mean titer and the standard deviation for each time point of the triplicate assays. Means with common letters are not significantly different at *P* ≤ 0.05. qRT-PCR failed to detect HMV2 RNA in normal cells and their supernatants without any treatment.

A viral growth kinetic analysis indicated that HMV2 could replicate in these three types of cells, but no CPE was observed in the process. Compared to Vero cells and BHK-21 cells, HMV2 replicated more efficiently and reached its peak after 10 days of infection in C6/36 cells. In addition, the HMV2 viral load reached its peak on the third and fifth days of infection in Vero cells and BHK-21 cells, respectively. Further, the viral load of HMV2 began to decrease gradually after reaching its peak in Vero cells and BHK-21 cells, whereas it could remain steady after reaching its peak in C6/36 cells (Fig. 3). In addition, HMV2 was not detected in the noninfected cells or supernatants.

### Genome-wide analysis of HMV2.

**FIG 4:**
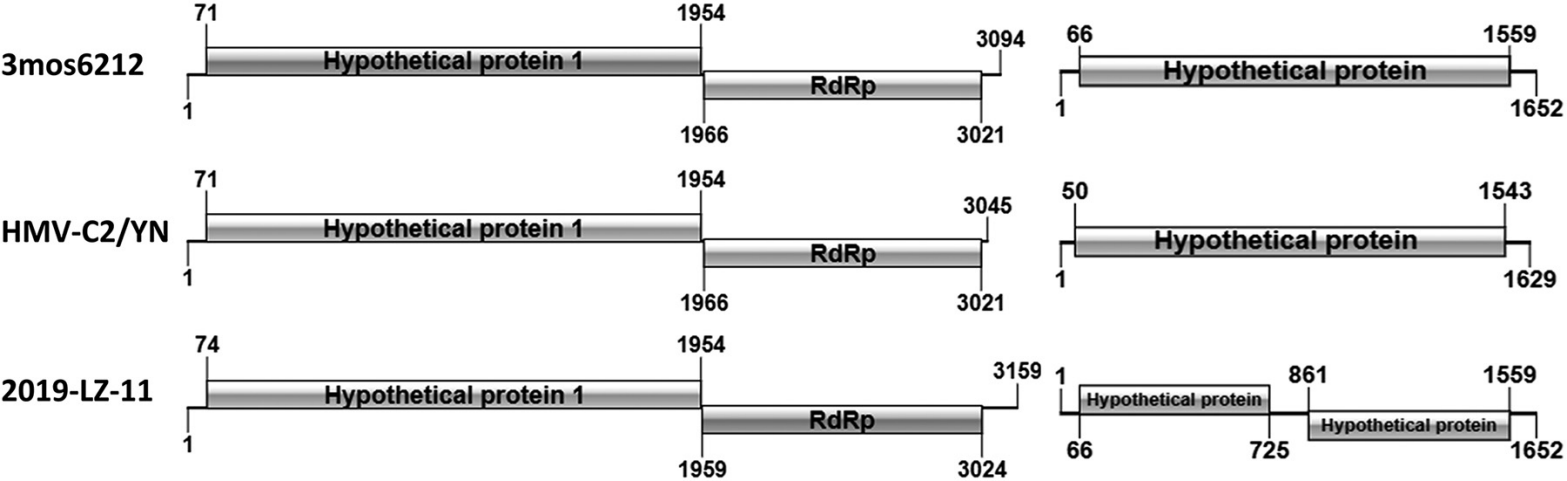
Structural features of two fragments of the genomic sequence of HMV2. The three genomic sequences of HMV2 were used to analyze and compare the genomic structure of HMV2. HMV2 strain 19-LZ-11 was obtained in this study, and HMV2 strains 3mos6212 and HMV-C2/YN were from the Hubei and Yunnan provinces, respectively.

The complete genome was successfully obtained from one mosquito pool that tested positive for HMV2. The HMV2 genome consisted of fragment 1, which was 3,159 nt in length, and fragment 2, which was 1,652 nt in length. Fragment 1 contained two open reading frames (ORFs), which were 1,884 and 1,056 nt in length, respectively. As shown in Fig. 4, ORF1 encoded hypothetical protein 1, and ORF2 encoded RNA-dependent RNA polymerase (RdRp). A protein prediction analysis showed that ORF1 manifested conserved domains for the peptidase S39 (608 to 982 nt) with 375 nt and the adventious gliding mobility protein GltJ (1,492 to 1,736 nt) with 245 nt, whereas ORF2 contained the NTP and nucleic acid binding sites of RdRp. Moreover, these two ORFs were separated by a 12-nt intergenic region, and the two ends of the fragment were 5′ and 3′ untranslated regions, which were 73 and 135 nt in length, respectively. Simultaneously, fragment 2 had a similar genomic structure to fragment 1. The fragment 2 of HMV2 was also composed of two ORFs, but these had lengths of 699 nt and 660 nt. The two ORFs were separated by a 136-nt intergenic region, and the 5′ and 3′ untranslated regions at both ends of the two ORFs were 66 and 93 nt in length, respectively. At the same time, the ORF1 of fragment 2 contained a 393 nt conserved domain of the viral coat protein (330 to 722 nt).

### Phylogenetic analysis.

**FIG 5:**
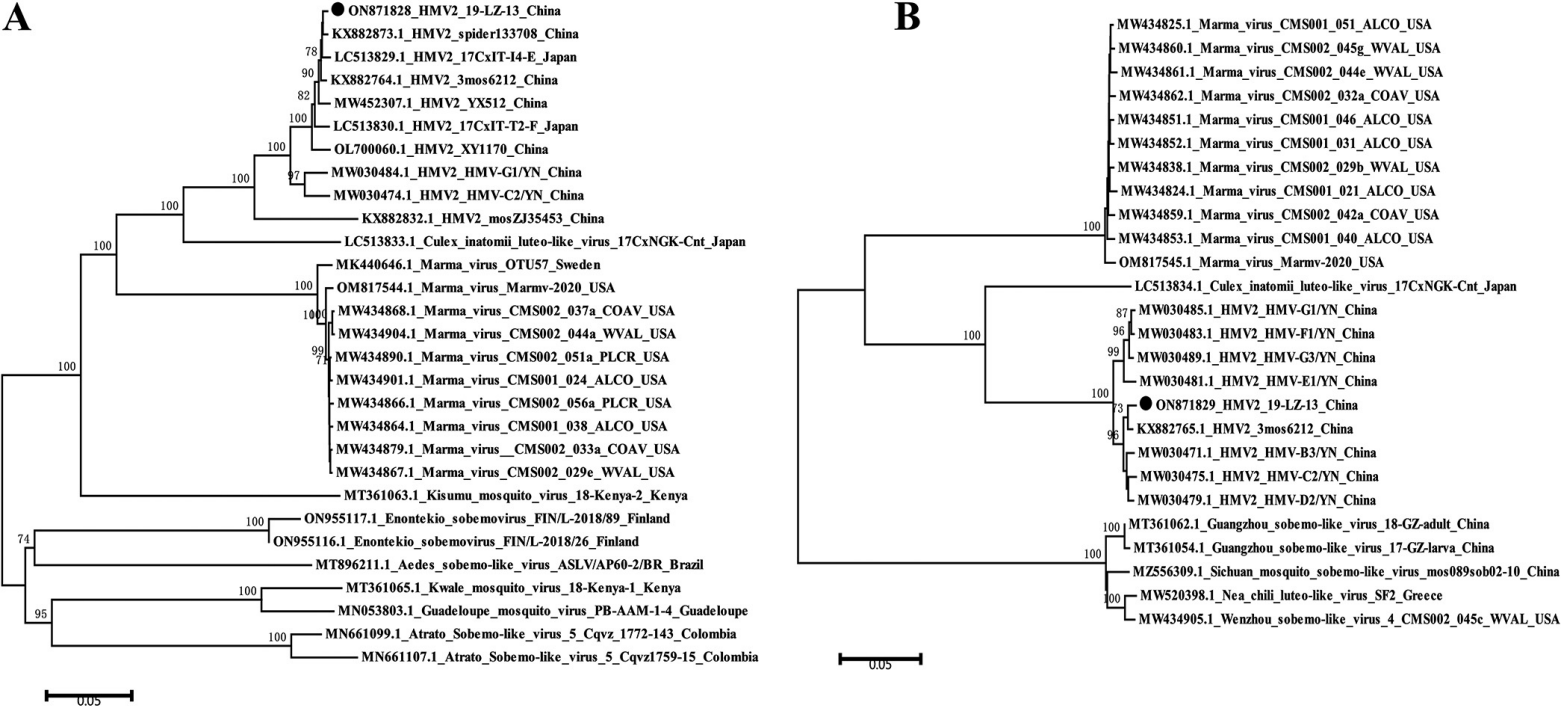
Phylogenetic analysis of the complete sequences of the HMV2 strains. The sequences that were identified from mosquitos in the current study are marked with black circles. The phylogenetic trees were constructed via the NJ method using MEGA 5.1, with 1,000 replicates and bootstrap values of >70% being regarded as significant. The numbers above the branches indicate the bootstrap values. (A) Phylogenetic analysis of HMV2, using the whole nucleotide sequences of fragment 1. (B) Phylogenetic analysis of HMV2, using the whole nucleotide sequences of fragment 2.

A nucleotide sequence identity analysis was performed among all of the available HMV2-related sequences, including the whole sequence of 2019-LZ-13 that was obtained in this study. The results showed that the fragment 1 of the HMV2 sequences that were obtained in this study were similar to the published HMV2 sequences in GenBank, with a nucleotide identity of 90.4% to 99.5%. On the other hand, fragment 2 of the HMV2 sequences had 96.4% to 99.2% identity with those that have been found. Phylogenetic trees were constructed, using a neighbor-joining method, based on the complete nucleic acid sequences of the 2 fragments to analyze the evolutionary relationship of HMV2 (Fig. 5A). All of the sequences could be classified into one of two major phylogenetic groups, with the first group containing Marma viruses that were identified from the USA, the HMV2 viruses from China and Japan, and the CiLLV from Japan, and the second group containing viruses that were sampled from Europe, Africa, and the Americas. Within the first group, all of the sequences could be further divided into two subgroups. All of the available HMV2 strains clustered together to form subgroup I, together with the CiLLV that were identified in Japan, and the Marma virus strain from the USA constituted subgroup II. The HMV2 strain in this study had a close evolutionary relationship with CiLLV in subgroup I, and it had a further evolutionary relationship with the Marma virus in subgroup II.

**FIG 6:**
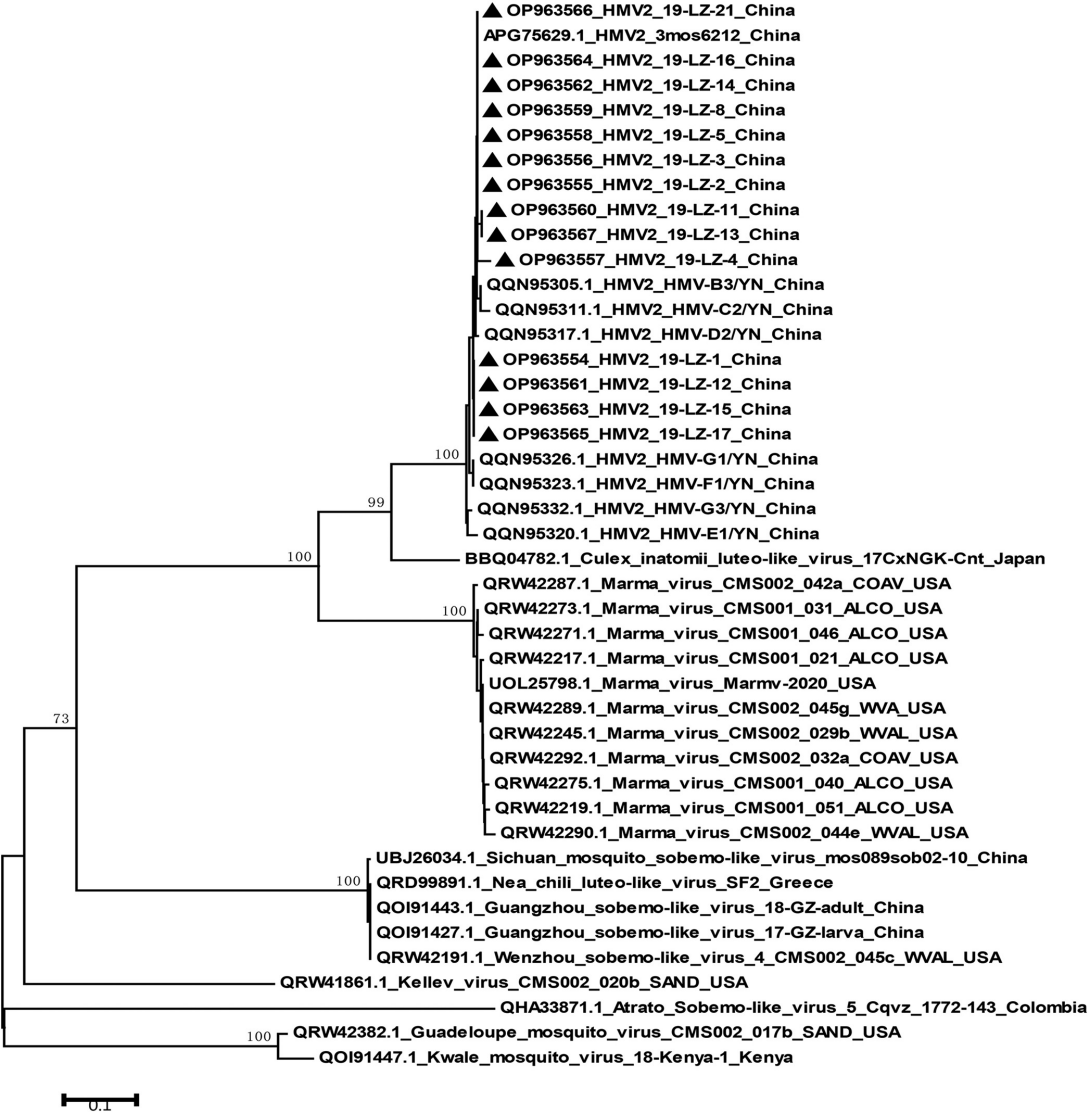
Phylogenetic analysis of the partial nucleotide sequences of HMV2 that were obtained in this study with HMV2-related virus sequences from GenBank. The phylogenetic tree was constructed based on partial sequences (1,002 bp) of the fragment 2 gene and was generated using the NJ method. The numbers above the branches indicate the bootstrap values. Black circles indicate the HMV2 sequences that were obtained in the current study.

In this study, the partial segment of HMV2 was amplified and sequenced from 14 of 28 qRT-PCR positive samples. A pairwise distance analysis demonstrated that all of the sequences had a nucleotide identity of 95.1% to 99.8% with each other, indicating that these sequences were highly similar. A phylogenetic tree was constructed based on 14 nucleotide sequences in this study, along with the corresponding sequences of the HMV2 and HMV2-related viruses that were retrieved from GenBank. As shown in Fig. 6, all of the HMV2 strains that were identified in this study clustered in phylogenetic subgroup I with the HMV2 strains from other parts of China. The composition of subgroup I clearly indicated that the HMV2 strains that have been identified to date around the world were highly homologous. These sequences of HMV2 clustered together and exhibited a close evolutionary relationship with CiLLV and a more distant evolutionary relationship with the Kwale mosquito virus from Kenya.

## DISCUSSION

HMV2 was considered to be a segmented RNA virus that was widely distributed in central and eastern China and in some regions of Japan, and it had formed a stable ecological cycle in these regions. Research on metagenomics showed that HMV2 had a wide host spectrum and could be carried by *Anopheles sinensis*, *Armigeres subalbatus*, Culex quinquefasciatus, and *Culex tritaeniorhynchus* (18). Additionally, research on HMV2 hosts had shown that the host of the virus was not only limited to the mosquito family but also included spiders, making the host spectrum of this virus broader than those of mosquito-specific viruses (16). The mosquito samples that were used in this study were mainly from livestock habitats, and the results largely reflected the survival of HMV2 in the natural environment. Therefore, in-depth studies of HMV2 were urgently needed to further understand the abundance, transmission, and diversity of the virus and its corresponding vectors in order to prevent a potentially significant public safety event caused by the virus.

HMV2 had been reported in Yunnan Province and Jiangsu Province, China, as well as in some areas of Japan, since HMV2 was identified in Hubei Province, China, in 2016. In this study, the HMV2 strains were detected for the first time in Shandong Province, China, which was previously an unrecognized area, suggesting that HMV2 may be widely distributed in eastern China. Meanwhile, HMV2 had been reported to be carried by four species of mosquitoes, including Culex quinquefasciatus and *Culex tritaeniorhynchus* (18), and our results were consistent with them. HMV2 was detected in samples of Culex quinquefasciatus and *Culex tritaeniorhynchus* that were collected from Shandong Province. The MIR value of HMV2 in the mosquitoes that were collected in this study was 1.00%. In comparison with a survey of the Tembusu virus from *Culex* mosquitoes from Shandong Province, China (19), the HMV2-positive rate in the mosquitoes in the present study was much lower. The difference in the positive rate may be due to sampling deviation or different calculation methods. In addition, we did not detect HMV2 in the mosquitoes that were collected in Weifang City. The reason for this may be related to the limitation of low sensitivity for the detection method of this novel virus or to the low carrying rate of HMV2 in the mosquitoes of this local area.

The electron micrograph of HMV2 showed that the virus particles were small and had no envelope. No CPE and cell disruption were found in C6/36 cells from the inoculation of the virus to the replication peak of the virus. Therefore, it was speculated that the release of virus did not destroy the cell membrane and may occur through intercellular bridges or cell fusion with adjacent cells.

C6/36, Vero, and BHK-21 cells were used to study the growth characteristics of HMV2. The results showed that all three kinds of cells supported the replication of HMV2. However, HMV2 replicated more efficiently in C6/36 cells than in Vero and BHK-21 cells, and the peak value of HMV2 replication in the C6/36 cells was significantly different from those in the other two types of cells. On day 10 of inoculation, HMV2 peaked in C6/36 cells and remained stable at its peak until the end of the culture. The virus replicated for a shorter period of time in Vero and BHK-21 cells, and it showed a decreasing trend after reaching the highest titer. The results of these studies imply that different cell lines may have an important influence on the replication of HMV2 and that C6/36 cells may be more suitable for the growth of HMV2. In addition, HMV2 may have similar growth characteristics to the Zika virus, and growth at 37°C may accelerate virus degradation, compared to 30°C (20). The optimal temperature for C6/36 cell growth was 30°C, whereas the optimal temperature for Vero and BHK-21 cells to grow was 37°C. Differences in temperature may cause the aforementioned to occur. Although no CPE was observed in the three types of cells, it could replicate in Vero cells and BHK-21 cells, suggesting that the virus was potentially pathogenic to mammals and possibly even to humans. At present, there are relatively few studies about HMV2, and its pathogenicity remains unclear. The potential threat of this virus to humans and animals requires continuous attention.

A comprehensive analysis of the amino acid sequences of all of the HMV2 strains was also performed. The results showed that fragment 1 of the 2019-LZ-13 strain from Shandong Province was significantly homologous in genomic structure to globally reported HMV2 strains that contain two ORFs. However, fragment 2 of the 2019-LZ-13 strain differed from the genomic structure of the previously identified HMV2 strains. Compared with the strain from Yunnan Province, the fragment 2 of the HMV2 strain from Shandong Province had a similar length, and its ORF also encoded a hypothetical protein. However, the protein underwent ORF disruption that was similar to that of the monkeypox virus (21). After sequence alignment, fragment 2 of the 2019-LZ-13 strain from Shandong Province, corresponding to the HMV-C2/YN strain from Yunnan Province, underwent a G→T transition at nt 723 and a T→A transition at nt 861. These results suggest that HMV2 may have been affected by the evasion of the mosquito immune system during infection, and this could have exerted some evolutionary pressure on the virus, thereby resulting in mutations in the codons of the coding region (22). Therefore, further experiments are needed to understand the structure and function of the proteins that are encoded by HMV2.

The complete sequence of the HMV2 obtained in this study was compared with all of the available HMV2 sequences in the GenBank database. The results of a pairwise distance analysis showed that all of the HMV2 sequences had >90% nucleotide homology to each other, indicating that there was significant similarity between these sequences from different locations. The phylogenetic analysis results of the two genomic fragments also validated this conclusion. All of the HMV2 strains clustered together, and they showed a close evolutionary relationship with CiLLV. These results suggested that HMV2 was evolutionarily conservative and highly adaptive to the local ecological environment.

A phylogenetic analysis of the partial segment of HMV2 indicated that the 14 HMV2 strains in this study were closely related to and highly homologous with strains that were identified elsewhere in China, implying that these HMV2 strains were highly conserved and showed the same evolutionary direction. The results also suggest that HMV2 strains from different geographical ranges may have originated from a common ancestor or may have genetically recombined in the same way. In addition, it had been suggested that some viruses infect hosts over wide geographic areas, possibly being introduced via being wind-blown, cyclonic action, or flying animals (13), and that HMV2 may have spread widely across different regions in this manner. Eventually, the exact mechanism of recombination evolution and the virus type of HMV2 could not be determined from these existing data, and in-depth studies need to be conducted to determine the origin and class of this virus.

In conclusion, our study provided important epidemiological evidence for the epidemic of HMV2 in Shandong Province. Here, we report the first isolation and molecular characteristics of this virus and enrich our knowledge of the distribution of HMV2 in China. Nevertheless, further studies are required to understand its pathogenic and epidemiological distribution characteristics as well as its transmission cycle in nature.

## MATERIALS AND METHODS

### Ethical approval and consent to participate.

The study protocol has been reviewed and approved by the Ethics Committee of Weifang Medical University.

### Mosquito collection and RNA extraction.

From July to August of 2019, mosquito samples were collected via mosquito traps at 16 locations in Weifang City and Yantai City, Shandong Province. The collected mosquito samples were immediately transferred to the laboratory and were stored in a refrigerator at −80°C. The collected mosquitoes were morphologically identified under a stereo microscope. Subsequently, PCR was used to amplify the cytochrome oxidase subunit I (*COI*) gene of the mosquitos, using the primers LCO1490: 5′-GGTCAACAAATCATAAAGATATTGG-3′ and HCO2198: 5′-TAAACTTCA GGGACAAAAAAATCA-3′ to verify the identification results. The identified mosquitoes were divided into different pools, according to their collection location and species, and there were no more than 50 mosquitoes in each pool. The samples in each pool were homogenized in 500 μL of RPMI medium. All of the homogenized samples were centrifuged at 10,000 rpm at 4°C for 5 min. Subsequently, 150 μL of homogenate liquid were taken from each pool and transferred to a corresponding 1.5 mL sterile centrifuge tube. The total RNA of each pool was extracted using a TIANamp Virus RNA Kit (Tiangen, China), according to the manufacturer’s instructions. The extracted nucleic acids were stored at −80°C for further use.

### Detection of HMV2 in mosquitos.

qRT-PCR was performed using a Qiagen One-Step RT-PCR Kit and HMV2-specific primer-probe sets to determine the presence of HMV2, according to the manufacturer’s instructions. The reaction was carried out in a volume of 25 μL, that contained 5 μL buffer 5×, 1 μL dNTP, 1 μL enzyme mix, 11.75 μL RNase-free water, 0.5 μL upstream primer and downstream primer (Table 1), 0.25 μL probe, and 5 μL RNA. The qRT-PCR was performed at 50°C for 30 min and 95°C for 15 min, and this was followed by 35 cycles of 94°C for 30 s, 55°C for 30 s, and 72°C for 1 min, with a final extension at 72°C for 10 min. The cycle threshold (Ct) value for a positive sample was set at 35 cycles. The primer-probe sets that were used in this study were designed and evaluated by Primer Express v3.0 (Applied Biosystems, USA). In short, alignments were performed with Clustal W (BioEdit v7.0.9), using the complete genomic sequences of HMV2 segment 2 that were downloaded from the GenBank database. After visual inspection, targeted genomic regions with high conservation were chosen. The primer-probe pairs were designed and appraised by Primer Express v3.0 (Applied Biosystems, USA), and a BLAST analysis was also performed to confirm the specificities of the primer-probe set.

**TABLE 1:** The primers and probe used for the identification of HMV2 in this study

Virus	Type	Primer/probe	Sequence (5′ to 3′)
HMV2	qRT-PCR	F	GTTCGTCGATGCTTGCTCC
		R	GGCTGCTGTGAGGTCGCA
		P	CTAGAGCAGTACAGACAGCGCTTCGGTCC
	Nested PCR	Outer-F	GAGTAACACAGGAATTAGCAGCGA
		Outer-R	CGTAAGAGCCCTCAAGTTCAGG
		Inner-F	GATCAGCCCGTCTAGAGTACCGT
		Inner-R	CTATCTGGTCGTTATGCTTGCCT

### Genome sequencing.

The total RNA of each qRT-PCR-positive mosquito pool was extracted, and reverse transcription PCR (RT-PCR) was performed with random hexamers to generate cDNA. After the cDNA was purified via ethanol precipitation, the sequencing library was constructed using a VAHTS Universal DNA Library Prep Kit for Illumina V3 (Vazyme, Nanjing, China), and the quality of the library was inspected using an Agilent 2100 Bioanalyzer (Agilent Technologies, Palo Alto, CA). The sequencing library was sequenced using an Illumina MiSeq system. The sequence data that were obtained via next-generation sequencing were edited and assembled using the MEGAHIT software package (version 1.2.9) to obtain the whole-genome sequence of HMV2.

In addition, the qRT-PCR-positive samples were also sequenced after nested PCR amplification. Nested PCR was performed, using HMV2-specific primers, to amplify the positive samples (Table 1). The nested PCR amplification results were purified via 1% agarose gel electrophoresis and were visualized using an imaging system (ChemiDoc Touch, USA). High-quality amplification products were sent to the Shanghai Sangon Biotech Company for Sanger sequencing.

### Cell culture.

Vero cells, BHK-21 cells, and C3/36 cells were used in this study. The C6/36 cells were grown in SF-9 medium containing 10% fetal bovine serum (FBS, Gibco) and 1% penicillin/streptomycin under conditions of 30°C and 3% CO_2_. Vero and BHK-21 cells were cultured with DMEM containing 10% fetal bovine serum (FBS, Gibco) and 1% penicillin/streptomycin at 37°C and 5% CO_2_.

### Virus isolation.

The mosquito homogenates of all of the qRT-PCR positive pools were filtered through a 0.22 μm microporous filter. Then, 100 μL of filtrate was extracted from each pool, inoculated on single-layer C6/36 cells with different holes in a six-well plate, and incubated at 30°C and 3% CO_2_ for 1 h. After 1 h of incubation, 2 mL of SF-9 medium containing 10% fetal bovine serum and 1% streptomycin were added to each well and cultured under the same conditions for 10 to 12 days. All of the samples were blindly passed through three generations. The cell supernatant of each generation was collected, and the cytopathic effect (CPE) was observed every day. The RNA in the collected supernatant was extracted, and qRT-PCR was performed to detect the presence of HMV2.

### Growth characteristics of the virus.

HMV2 was inoculated into three different cells to understand its viral growth characteristics. Normal cells without any treatment were used as controls. Vero, BHK-21, and C6/36 cells that were grown in T75 flask plates were infected with the virus. After 24 h of infection, the culture medium of the culture flask was discarded, and fresh medium was added. Then, the cells were observed daily for CPE. The cell culture supernatant was collected every 48 h and stored at −80°C for further analysis. Viral RNA was extracted and detected via qRT-PCR, and the virus concentration was calculated based on the standard curve, according to the generated Ct values.

### Electron microscopy.

As previously described, the C6/36 cells in the T75 culture flasks were infected with HMV2, and the supernatant was collected 15 days after infection. qRT-PCR was used to identify and quantify the HMV2 in the supernatant. Negative staining was used for the electron microscopic observation of the virus. First, to purify the virus in the supernatant, the cell supernatant was centrifuged at 4°C for 30 min at 5,000 rpm to remove cell debris. Then, ultracentrifugation through a sucrose pad was performed at 4°C at 40,000 rpm for 3.5 h. After the ultracentrifugation was completed, the supernatant was discarded, and a buffer salt solution was added to the tube to resuspend the virus particles. Finally, the virus was stained with phosphotungstic acid and observed under an electron microscope.

### Data analysis.

The prevalence of HMV2 in mosquitoes was expressed as the minimum infection rate (MIR), which was calculated by dividing the number of positive pools by the total number of mosquitoes tested. The statistical analysis of the data was performed by using the GraphPad Prism software package (version 8.0). Fisher’s exact test was performed to assess the statistical differences in the positive rates. *P* values of <0.05 were considered to be indicative of a statistically significant result. The complete nucleotide sequence of HMV2 was analyzed using NCBI’s ORF finder tool to identify the open reading frame. The available sequences of related viruses were obtained from the GenBank database via BLASTN, and they were compared with the complete sequence of HMV2 that was obtained in this study. The phylogenetic tree was constructed via the neighbor-joining method, and the reliability of the tree was determined via the bootstrap method (1,000 repetitions). Bootstrap values of >70% were considered to be significant. At the same time, the evolutionary distance of the HMV2 sequence that was obtained in this study was analyzed using the DNA Star software package (Lasergene 7.0, USA).

### Data availability.

The data are available upon request by email to the corresponding author. All of the nucleotide sequences were deposited in GenBank under the accession numbers ON871828 to ON871829 and OP963554 to OP963567.
